# Transcriptional Regulation of TMP21 by NFAT

**DOI:** 10.1186/1750-1326-6-21

**Published:** 2011-03-07

**Authors:** Shengchun Liu, Si Zhang, Kelley Bromley-Brits, Fang Cai, Weihui Zhou, Kun Xia, Jill Mittelholtz, Weihong Song

**Affiliations:** 1 Department of Surgery, The First Affiliated Hospital, Chongqing University of Medical Sciences, Chongqing 410006, China; 2 Townsend Family Laboratories, Department of Psychiatry, Brain Research Center, Graduate Program in Neuroscience, The University of British Columbia, 2255 Wesbrook Mall, Vancouver, BC V6T 1Z3, Canada; 3 The State Key Lab of Medical Genetics of China, Central South University, Changsha, Hunan 410078, China

## Abstract

**Background:**

TMP21 is a member of the p24 cargo protein family, which is involved in protein transport between the Golgi apparatus and ER. Alzheimer's Disease (AD) is the most common neurodegenerative disorder leading to dementia and deposition of amyloid β protein (Aβ) is the pathological feature of AD pathogenesis. Knockdown of TMP21 expression by siRNA causes a sharp increase in Aβ production; however the underlying mechanism by which TMP21 regulates Aβ generation is unknown, and human TMP21 gene expression regulation has not yet been studied.

**Results:**

In this report we have cloned a 3.3-kb fragment upstream of the human TMP21 gene. The transcription start site (TSS) of the human TMP21 gene was identified. A series of nested deletions of the 5' flanking region of the human TMP21 gene were subcloned into the pGL3-basic luciferase reporter plasmid. We identified the -120 to +2 region as containing the minimal sequence necessary for TMP21 gene promoter activity. Gel shift assays revealed that the human TMP21 gene promoter contains NFAT response elements. Expression of NFAT increased TMP21 gene expression and inhibition of NFAT by siRNA reduced TMP21 gene expression.

**Conclusion:**

NFAT plays a very important role in the regulation of human TMP21 gene expression. This study demonstrates that the human TMP21 gene expression is transcriptionally regulated by NFAT signaling.

## Background

Alzheimer's disease is the most common neurodegenerative disorder leading to dementia. Deposition of amyloid β protein (Aβ) in the brain is one of the hallmarks of AD pathogenesis. Aβ is generated from a larger β-amyloid precursor protein (APP) by sequential cleavages of β-secretase and γ-secretase [1-4]. Beta-site APP cleaving enzyme 1 (BACE1) is the β-secretase *in vivo *[5,6], and γ-secretase activity is catalyzed by a presenilin (PS)-containing macromolecular complex [7]. This high molecular weight complex also requires nicastrin (Nct) [8], anterior pharynx-defective 1 (Aph-1) [9-11], and presenilin enhancer 2 (Pen-2) [9,10] for its enzymatic activity [7,11-13]. CD147 was also found to closely associate with the γ-secretase complex [14]. Despite robust expression of the APP gene resulting in a high level of APP protein *in vivo*, Aβ production through the amyloidogenic pathway of APP processing is a rare occurrence under normal conditions [15,16].

Missense mutations in *PS1 *and *PS2 *are a major cause of early-onset familial AD (FAD). PS mutant forms have been shown to increase γ-secretase activity, resulting in elevated Aβ42 production [17-19]. In addition to its pathogenic γ-secretase activity, the PS complex can cleave Notch [20,21] at a separate ε-cleavage site which may be involved in learning and memory. Intramembranous cleavage of Notch to release the Notch intracellular domain (NICD) is inhibited in PS1-deficient cells [8,20,22-24]. In addition to APP and Notch, the substrates for γ-secretase include Jagged, Delta, E-cadherin, ErbB-4, Nectin-1α, CD44 and LRP. The ε- and γ-secretase activities of the presenilin complex seem to be independently regulated, as certain γ-secretase inhibitors can affect APP cleavage without affecting Notch cleavage, and presenilin mutations can increase Aβ-42 production while decreasing Notch cleavage [25,26].

Human TMP21, a member of the p24 protein family, is a type I transmembrane protein with a large luminal domain [27]. It is ubiquitously transcribed with higher protein levels in the pancreas and intestines. The cytoplasmic tails of p24 family interact with coatomer, a major component of coat protein complex I (COP-I), to facilitate transport between the ER and Golgi [28-30]. TMP21 cycles through the early secretory pathway between the intermediate and *cis*-Golgi compartments [31-33], and is necessary for proper organization of the Golgi apparatus [34,35]. TMP21 also plays an essential and non-redundant role in the earliest stages of mammalian development [36]. Recently, TMP21 was identified as a new member of the PS-associated complex [37]. Our recent study showed that TMP21 is ubiquitinated and that degradation of TMP21, as with the other PS-associated γ-secretase complex members, is mediated by the ubiquitin-proteasome pathway [38]. Interestingly, TMP21 has been shown to regulate Aβ generation but not Notch processing, suggesting that TMP21 selectively regulates γ-secretase, but not ε-secretase, activity [37].

Gene expression requires tightly controlled gene transcription regulated by interaction between *cis*-acting elements in the promoter region and transcription factors. It is well known that the nuclear factor of activated T cells (NFAT)/calcineurin family play a central role in inducible gene transcription in various signaling pathways, regulating cell differentiation, development, adaptation, immune system response, inflammatory, adipocyte metabolism, and lipolysis, as well as carcinogenesis [39-42]. There are a few NFAT family isoforms in different tissue. NFAT localization in the nucleus is dependent on the import-export balance between the activity of Ca2+/calmodulin-dependent phosphatase, calcinurin, and the activity of serine/threonine kinase [43]. NFAT signaling is pivotal during embryogenesis for cardiovascular development [44], and affects the cytokine and immunoregulatory gene transcriptional activator in T cells, as well as various other physiological activities beyond the immune system. It is also involved in regulation of cell growth, differentiation, and cell cycle progression in diverse cell types after birth [45,46].

NFAT proteins are phosphorylated and constitutively expressed in resting cells [47,48]. Phosphorylated NFAT normally resides in the cytoplasm and has low affinity for DNA binding [49,50]. The anastomosis of cell surface receptors to the calcium-signaling pathway activates phospholipase C-γ, causing phosphatidylinosito-4,5-biphosphate, a plasma membrane component, to be hydrolyzed, producing cytosolic inosito-1,4,5-trisphosphate (IP3) and membrane-bound diacylglycerol. IP3 induces calcium release from the ER, stimulating calcium-activated calcium channels on the plasma membrane to open, which then maintains the increased level of intracellular calcium. The ratio of calcium/calmodulin triggers Ser/Thr-phosphatase calcineurin (CaN), which can dephosphorylate NFAT [51]. Dephosphorylated NFAT protein can be translocated to the nucleus and has a high affinity for DNA binding, inducing NFAT-dependent gene transcription [48,52-54]. The calcineurin inhibitors CsA or FK506 can block NFAT activation. The NFAT binding site in the promoter of target genes has a 9 bp consensus sequence (A/T)GGAAA(A/N)(A/T/C)N [47]. The calcineurin inhibitors CsA or FK506 can block NFAT activation. The mechanism underlying the transcriptional regulation of human TMP21 and its role in neuropsychiatric disorder pathogenesis is unknown. In order to study the molecular mechanism of human TMP21 gene transcription regulation, we cloned and functionally characterized the human TMP21 gene promoter. We found that transcription factor NFAT plays a pivotal role in regulation of TMP21 gene transcription.

## Results

### Cloning the human TMP21 gene promoter and mapping its transcription start site

**Figure 1:**
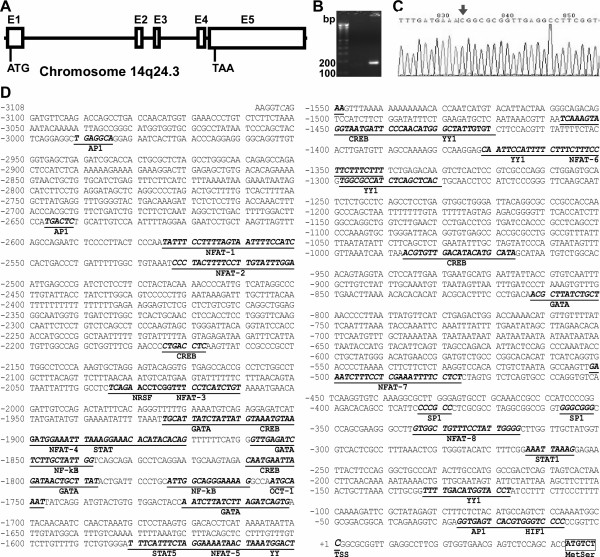
**Sequence features of the human TMP21 gene promoter and identification of the transcription start site**. (**A**) The genomic organization of human TMP21 gene on Chromosome 14q24.3. E represents exon. ATG is the translation start codon and TAA the stop codon. (**B**) RLM-RACE experiment was performed to map the *TMP21 *transcription start site. Neuronal RNA was extracted by TRI-Reagent from SH-SY5Y cells. Total RNA was treated with Calf Intestine Alkaline Phosphatase to remove free 5'-phosphates and the RNA was then treated with Tobacco Acid Pyrophosphatase (TAP) to remove the cap structure from full-length mRNA, leaving a 5'-monophosphate. A 45 base RNA Adapter oligonucleotide was ligated to the RNA population using T4 RNA ligase. A random-primed reverse transcription reaction and nested PCR then amplified the 5' end of a specific transcript. The product was analyzed on a 1.5% agarose gel. (**C**) The PCR product was cloned into pcDNA4-mycHis at BamHI and XhoI sites. DNA sequencing was performed to identify the insert sequence. The first base pair after the adapter was the transcription start site (TSS). The arrow indicates the TSS. (**D**) The nucleotide sequence of the human TMP21 gene promoter. A 3333-bp fragment of the 5' flanking region of the human TMP21 gene was isolated from a human BAC genomic clone and sequenced by the primer walking strategy. The cytosine +1 represents the TSS mapped by RLM-RACE. The putative transcription factor binding sites are underlined in bold face. Amino acid codons were boxed in boldface. Genbank™accession number is JF694939.

Human TMP21 is ubiquitously transcribed with higher protein levels in the pancreas and intestines. The human *TMP21 *gene is located on chromosome 14q24.3 and spans 45,179 base pairs. It has five exons and encodes for a 21-kD protein of 219 amino acids (Figure 1A). To examine its transcriptional regulation, 3333 bp of the 5' flanking region of the human *TMP21 *gene was cloned from human genomic DNA. To determine the transcription start site of TMP21, RNA Ligase Mediated Rapid Amplification of cDNA Ends (RLM-RACE) assay was performed. A 182 bp major cDNA product from the nested-PCR was amplified and cloned into pcDNA4 vector (Figure 1B). DNA sequencing indicates that the major transcription start site is located at 43 bp upstream of the translation start site ATG. This transcription initiation site is designated as +1 and begins with cytosine (Figure 1C). Sequence analysis and a computer-based transcription factor binding site search (MatInspector 2.2, Genomartrix, Oakland, CA, USA) reveals that the human TMP21 gene has a complex transcriptional unit. The human TMP21 gene promoter lacks typical CAAT and TATA boxes and contains several putative regulatory elements, such as AP1, SP1, CREB, NFAT, HIF-1, GATA as well as STAT (Figure 1D).

### Functional characterization of the TMP21 promoter

**Figure 2:**
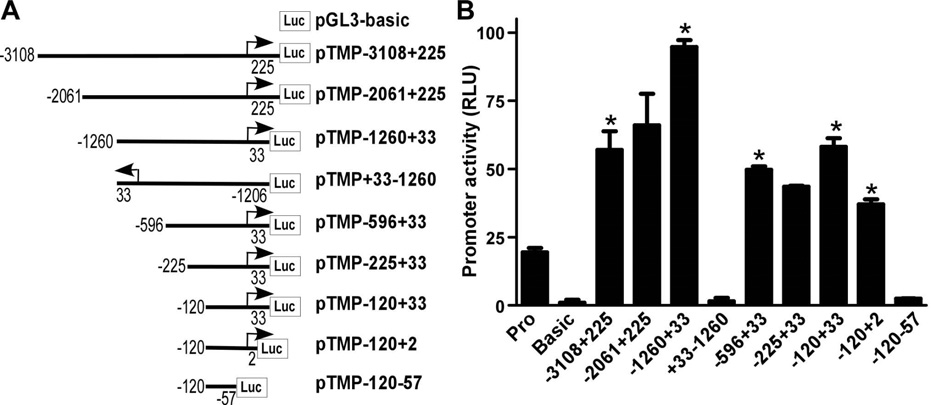
**Deletion analysis of the human TMP21 gene promoter**. **(A) **Schematic diagram of the TMP21 promoter constructs consisting of the 5' flanking region with serial deletions cloned into the pGL3-basic vector. Arrow shows the direction of transcription. The numbers represent the end points of each construct. The deletion plasmids were confirmed by sequencing and restriction enzyme digestion. **(B) **The plasmid constructs were co-transfected with pRLuc into HEK293 and SH-SY5Y cells by Lipofectamine 2000^R^. After 48 hour transfections the cells were harvested and luciferase activity was measured with a luminometer and expressed in Relative Luciferase Units (RLU). *Renilla *luciferase activity was used to normalize for transfection efficiency. The values represent means ± SEM. *, P < 0.001 by analysis of variance with the post hoc Newman-Keuls test.

To determine whether the 3.3-kb fragment from -3108 to +225 bp of the *TMP21 *5' flanking region contains the human *TMP21 *promoter, the pTMP-3108+225 plasmid was constructed to contain this fragment upstream of a luciferase reporter gene in the promoterless plasmid pGL3-basic. The pGL3-basic vector lacks a eukaryotic promoter and enhancer sequences upstream of the reporter luciferase gene. Expression of luciferase in cells transfected with this plasmid depends on a functional promoter upstream of the luciferase gene. Plasmid DNA was transfected into HEK293 cells and luciferase activity was measured with a luminometer to determine promoter activity. Promoterless empty vector pGL3-basic was used as negative control and CMV promoter-luciferase construct pGL3-promoter was transfected as a positive control. Compared with cells transfected with pGL3-basic or pGL3-promoter controls, cells transfected with pTMP-3108+225 had significantly higher luciferase activity (57.03 ±6.83 RLU, P < 0.001) (Figure 2B). This result indicated that the 3.3 kb 5' flanking region of the TMP21 gene contains the functional promoter for human TMP21 gene transcription.

To functionally analyze the TMP21 promoter and identify the minimal promoter region needed for TMP21 gene expression, a series of deletions of the 5' flanking fragments of *TMP21 *were subcloned into pGL3-basic (Figure 2A). Deletion of 1047 bp upstream from pTMP-3018+225 had no significant effect on the luciferase activity of cells transfected with plasmid pTMP-2061+225 (66.02 ± 11.51RLU) (P > 0.05 relative to pTMP3018+225). However, further deletions (pTMP-1260+33) had significantly higher promoter activity (94.70 ± 2.54 RLU, P < 0.005), suggesting the deleted region might contain inhibitory *cis-acting *elements. Inserting the -1260+33 fragment in the reverse direction upstream of luciferase reporter gene (plasmid pTMP+33-1260) resulted in little luciferase expression. These data further supported that the 5' flanking region contains the functional human TMP21 gene promoter.

To identify the minimal region containing TMP21 promoter activity, further deletion plasmids were constructed. Plasmid pTMP-596+33, pTMP-225+33 and pTMP-120+33 had luciferase activity at 49.78 ± 1.16, 43.51 ± 0.41, and 58.16 ± 3.11 RLU, respectively (Figure 2B). A 31 bp deletion from the 3' end of the -120 to +33 bp fragment significantly reduced the luciferase activity from 58.16 ± 3.11 RLU in pTMP-120+33 to 37.07 ± 1.85 RLU in pTMP-120+2 (P < 0.005). However, a further 58 bp 3' deletion resulted in little luciferase activity in pTMP-120-57 (P < 0.001) (Figure 2B). The results indicate that the 5' flanking region from -120 to +2 bp, containing the transcription initiation site, is the minimal promoter region necessary for basal transcription of *TMP21*, and the fragment from -120 to -57, lacking the transcription start site, has no promoter activation ability.

### The human TMP21 gene promoter contains NFAT cis-acting response elements

**Figure 3:**
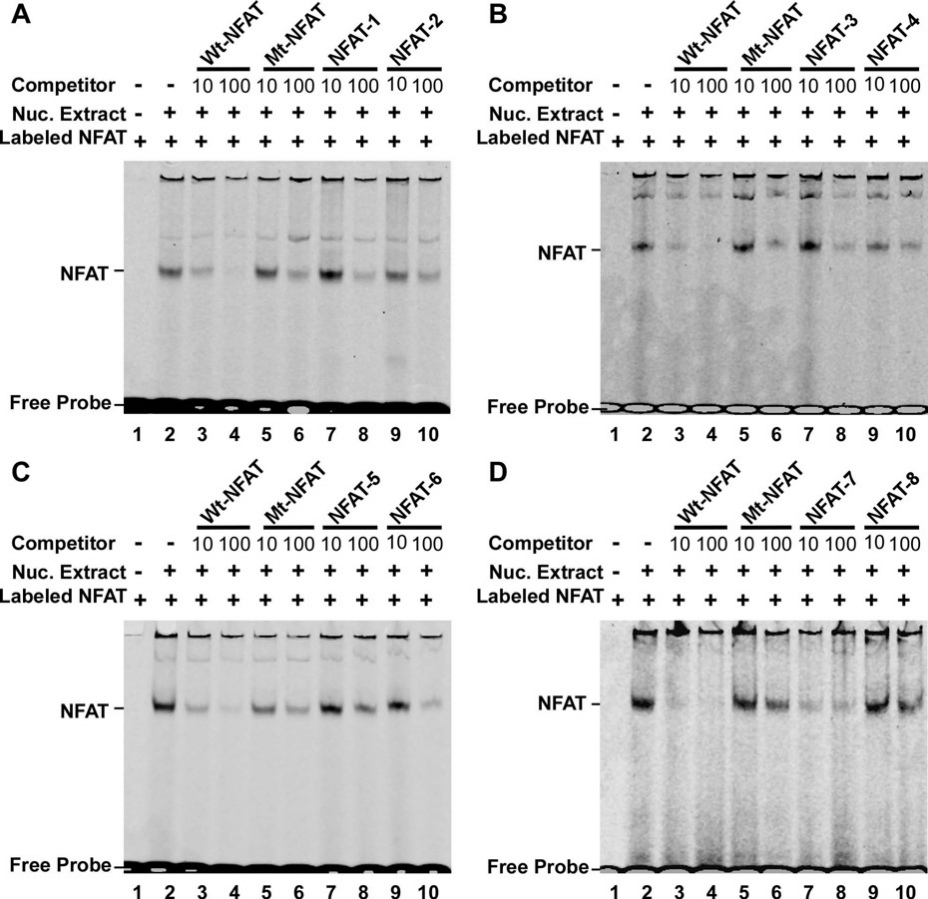
**Gel mobility shift assay for the TMP21 gene promoter**. Gel shift assays were performed as described in the Material and Methods. Double stranded NFAT oligonucleotide probes labeled wuth IRDye-680 were applied. Lane 1 is labeled probe alone without nuclear extract. Incubation with nuclear extracts retarded the migration rate of the labeled probe, forming a new shifted DNA-protein complex band (lane 2). Competition assays were performed by further adding different concentrations of molar excess of unlabeled competition oligonucleotides, consensus NFAT (lane 3, 4), binding sequence mutant NFAT (lane 5, 6) and TMP21 NFAT elements (lane 7, 8, 9, 10).

NFAT signaling regulates gene transcription by translocation of activated dephosphorylated NFAT into nucleus to bind an NFAT response element in the promoter of target genes. The NFAT binding element contains a 9 bp consensus sequence (A/T)GGAAA(A/N)(A/T/C)N [49]. Sequence analysis indicates that there are eight putative NFAT binding sites in the 3.1 kb promoter region of the human TMP21 gene (Figure 1B). To investigate whether those putative NFAT elements are functional NFAT binding sites, gel shift assays were performed. A 12-bp consensus double-stranded NFAT oligonucleotide probe was synthesized and end labeled with IRDye-680 (Li-COR, Biosciences). A shifted protein-DNA complex band was detected after incubating the labeled NFAT probe with HEK293 nuclear extract (Figure 3, lane2 of panel ABCD). The intensity of the shifted band was significantly reduced by adding 10 fold excess of unlabeled NFAT consensus competition oligonucleotides (Wt-NFAT), and the band was almost entirely abolished by applying a 100-fold excess of unlabeled NFAT consensus oligonucleotides (Figure 3, lane 3,4 of panel ABCD). Applying a 10-fold excess of mutant NFAT consensus oligonucleotides (Mt-NFAT), which contain a binding site mutation, had no competing effect on the NFAT protein-DNA binding complex shifted band (Figure 3, Lane 5 of Panel ABCD). 100-fold Mt-NFAT oligos could show some non-specific competition (Lane 6). Only a 10-fold and 100-fold excess of the 7th putative NFAT response element (TMP21-NFAT-7) could markedly reduce the intensity and completely abolish the signal of the shifted bands, respectively, when compared to preincubation of the 8 unlabeled TMP21-NFAT probes with HEK293 nuclear extract, (Figure 3D). This result indicated that a NFAT binding site is located at -502 bp to -476 bp of the human TMP21 promoter region.

**Figure 4:**
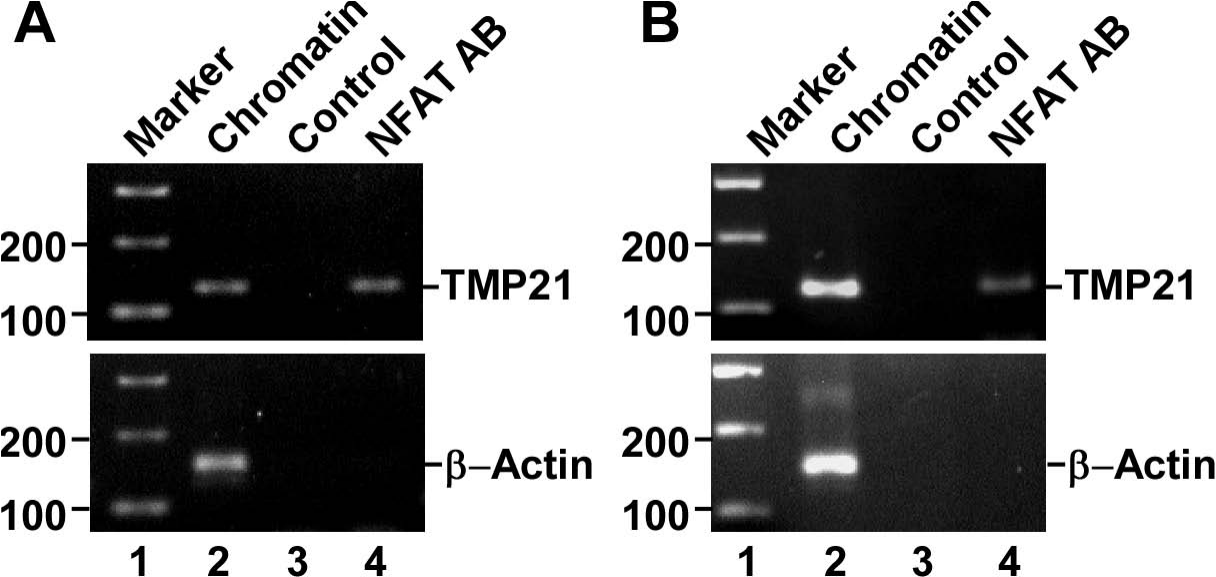
**ChIP assay to show that NFAT proteins are associated with human TMP21 promoter**. ChIP assay was performed as described in the Material and Methods. The chromatin was isolated HEK293 (**A**) or SH-SY5Y cells (**B**), and then sheared from the cells treated with cross-link reagent formaldehyde. For isolation of NFAT binding complex, the chromatin solution was incubated with NFAT antibody (Santa Cruz). PCR amplifications were performed using TMP21 promoter-specific primers, with samples from the sheared chromatin alone (lane 2), non-NFAT antibody control (lane 3) and NFAT-immunoprecipitating chromatin sample (lane 4). β-actin was used as internal control.

To investigate the interaction between transcription factor NFAT and the human TMP21 promoter in HEK293 (Figure 4A) and SH-SY5Y (Figure 4B) cells, chromatin immunoprecipitation (ChIP) assay was performed. As positive control, TMP21 promoter and β-actin DNA fragment were amplified from the sheared chromatin samples (lane 2). Only TMP21 promoter fragment can be amplified from the sheared chromatin sample immunoprecipitated by NFAT antibody (lane 4, but not from the chromatin sample incubating with non-NFAT antibody solution control (lane 3). No β-actin DNA fragment was pulled down from those two samples either. The results clearly showed that NFAT proteins are associated with TMP21 promoter region in both HEK293 and SH-SY5Y cells.

### NFAT expression increases and pyrrolidine dithiocarbamates (PDTC) decreases human TMP21 promoter activity

**Figure 5:**
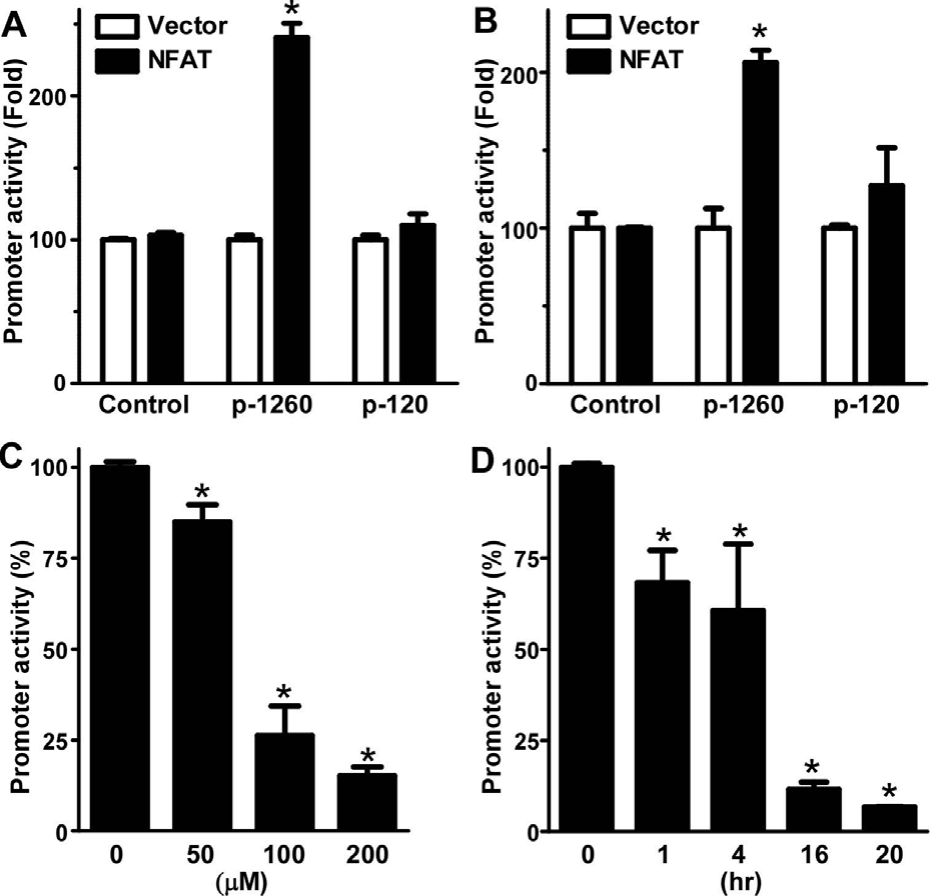
**Regulation of TMP21 promoter activity by NFAT and PDTC**. Transcriptional activation of the TMP21 promoter is potentiated by NFAT. Empty vector and TMP21 promoter plasmid pTMP-1260+33 or pTMP-120+33 were cotransfected with NFAT expression plasmid pHA-NFAT1 into HEK293 cells **(A) **or N2a cells **(B)**. Overexpression of NFAT significantly increased pTMP21-1260+33 or promoter activity and had no effect on pTMP-120+33 or control plasmid in both HEK293 and N2a cells. n = 4, * p < 0.0001. **(C, D) **Inhibition of TMP21 promoter activity by PDTC. TMP21 promoter construct pTMP21-1260+33 and pRluc were cotransfected into HEK293 and N2a cells. After 24 h cells were treated with varying dosages of PDTC for 16 hours **(C) **or at 100uM for varying times **(D)**. Cells were harvested at the same time for luciferase assay. *Renilla *luciferase activity was used to normalize for transfection efficiency. The values represent means ± SEM relative to control promoter activity. *, P < 0.001 relative to control by ANOVA with the post hoc Newman-Keuls test.

To examine the role of NFAT signaling in the regulation of human TMP21 transcription and expression, we first examined TMP21 promoter activity in cells overexpressing NFAT protein. Either pTMP-1260+33 or pGL3 promoter were co-transfected with NFAT expression plasmid or empty vector into HEK293 or N2a cells. pRLuc was also transfected for transfection efficiency normalization. The cells were harvested with lysis buffer 48 later after transfection and luciferase activity was measured. The luciferase activity of pTMP-1260+33 was significantly increased by 2.40-fold in cells co-transfected with NFAT expression plasmid (P < 0.001) (Figure 5A). To further demonstrate that NFAT site in TMP21 promoter mediates NFAT's effect on TMP21 expression, pTMP-120+33 was also co-transfected with NFAT expression plasmid or empty vector. pTMP-120+33 promoter plasmid lacks NFAT binding site and our results showed that NFAT overexpression had no significant effect on its luciferase activity (P > 0.05) (Figure 5A). These results indicate that human TMP21 gene promoter activity can be significantly up-regulated by NFAT over-expression via its NFAT response element.

Pyrrolidine dithiocarbamates (PDTC) is an antioxidant compound which strongly inhibits NF-κB and activates AP-1 signaling. PDTC was also shown to have a strong inhibitory effect on NFAT signaling and its downstream gene transcription [55]. To further examine NFAT's effect on TMP21 transcription, HEK293 and N2a were transfected with plasmid pTMP-596+33. The cells were then treated with 100 μM PDTC for varying times and doses of PDTC for 24 hours. Cell lysates were assayed for luciferase activity. PDTC treatment significantly reduced TMP21 promoter activity. Addition of 50, 100, 200 uM of PDTC for 16 hours decreased promoter activity to 84.99 ± 4.72%, 26.25 ± 8.11% and 15.15 ± 2.48%, respectively (P < 0.001) (Figure 5C). 100 μM PDTC treatment for 1, 4, 16 and 20 hours reduced TMP21 promoter activity to 68.32 ± 8.84%, 60.67 ± 18.23% and 11.63 ± 2.01% and 6.80 ± 0.06%, respectively (P < 0.001) (Figure 5D). These data demonstrated that NFAT inhibitor PDTC can inhibit human TMP21 promoter activation in a time- and dosage-dependent manner.

### Regulation of TMP21 expression by NFAT activity

**Figure 6:**
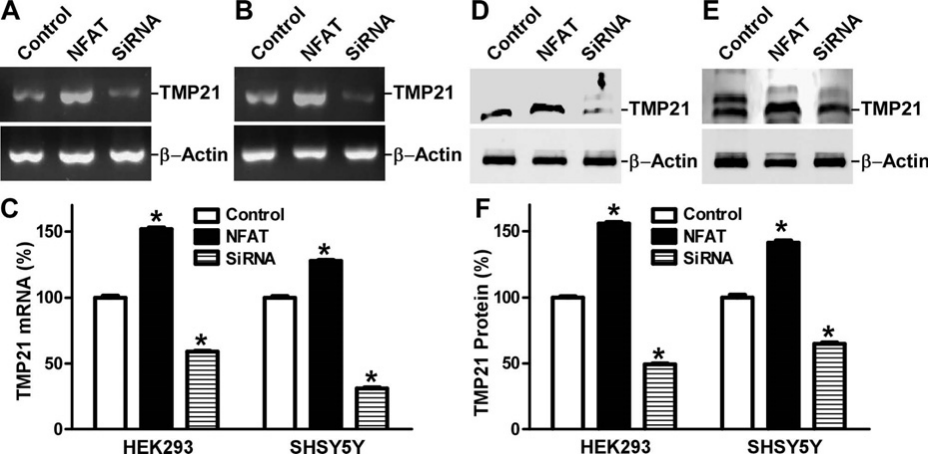
**NFAT facilitates TMP21 gene expression at both the mRNA and protein level**. HEK293 **(A) **and SH-SY5Y **(B) **cells were transfected with NFAT expression plasmid or NFAT siRNA (sc-29412, Santa Cruz Biotechnology, Inc.). Total RNA was extracted. Semi quantitative RT-PCR was performed to detect the mRNA levels of TMP21 and β-actin. Specific TMP21 and β-actin coding sequence primers were used to amplify the TMP21 and β-actin cDNA, as described in Materials and Methods. The RT-PCR products were analyzed on 1% agarose gels. β-actin was used as internal control. **(C) **The ratio of TMP21 to β-Actin mRNA was quantitated by Kodak Image Analysis. Shown is the Mean+S.E.M., and n = 4. *p < 0.001 relative to controls by ANOVA with post-hoc Newmann-Keuls test. **(D and E) **Western blot assay was then performed to analyze cell lysates from NFAT-transfected cells and NFAT siRNA on 16% Tris-Glycine gel. TMP21 protein was detected using rabbit anti-mouse anti-TMP21 ployclonal antibody T21 and β-actin was used as an internal protein control. Overexpression of NFAT increased TMP21 protein generation and NFAT siRNA decreased the protein levels in HEK293 **(D) **and SH-SY5Y **(E) **cells. **(F) **Quantitative analysis of the generation of TMP21. Values are Means ± S.E.M. and n = 4. The protein levels are expressed as a percentage of the levels in control cells. * p < 0.001 relative to controls by ANOVA with post-hoc Newmann-Keuls test.

To examine the effect of NFAT on endogenous TMP21 mRNA and protein levels, HEK293 and SH-SY5Y cells were transfected with NFAT expression plasmid and NFAT-specific siRNA. The samples were analyzed by semi-quantitative RT-PCR and Western blot with β-actin levels as the internal control. RT-PCR showed that NFAT expression significantly increased endogenous TMP21 mRNA levels (151.73 ± 1.36%, P < 0.001), while knockdown of NFAT expression by siRNA markedly reduced endogenous TMP21mRNA levels (58.94 ± 0.63%, P < 0.001) in HEK293 cells (Figure 6A and 6C). Similar results were also observed in SH-SY5Y cells: NFAT overexpression increased TMP21 mRNA levels to 128.00 ± 0.66% and NFAT siRNA reduced the levels to 30.98 ± 0.34% (P < 0.001 relative to control) (Figure 6B and 6C). Consistent with the transcription data, Western blot analysis showed that TMP21 protein levels were significantly increased by NFAT expression in HEK293 cells (155.78 ± 1.075%) (Figure 6D and 6F) and SH-SY5Y cells (141.62 ± 1.64%) (Figure 6E and 6F), and knockdown of NFAT expression markedly decreased TMP21 protein levels in HEK293 (49.29 ± 0.64%) (Figure 6D) and SH-SY5Y cells (64.83 ± 0.96%) (Figure 6E), respectively (P < 0.001 relative to control) (Figure 6F). Taken together, these results clearly demonstrate that NFAT signaling regulates human TMP21 gene expression.

## Discussion

Beta-secretase cleavage of APP by BACE1 produces APP C-terminal fragment C99, which can be cleaved by the γ-secretase complex to form Aβ and subsequently aggregate to form classic AD plaques. The γ-secretase complex requires PS1, Nct-1, Aph-1, and Pen-2 for its enzymatic activity [7]. Recently, TMP21 was identified as a member of the γ-secretase complex [37]. Knockdown of TMP21 with siRNA increased γ-secretase activity and Aβ production without altering the relative amounts of γ-secretase complex components or APP substrate, suggesting that TMP21 is a selective regulator of γ-secretase [37]. TMP21 is a type I transmembrane protein involved in ER/Golgi transport [27]. Interestingly, the cDNA sequence of TMP21 is similar to cDNA clone S31iii125, which was identified within the AD3 locus on chromosome 14q24.3, which is associated with aggressive, early-onset AD [56]. This, coupled with its role as a selective regulator of γ-secretase activity, strongly suggests that TMP21 may play an as of yet undetermined role in AD pathogenesis.

The TMP21 gene is prolifically expressed in the pancreas, nervous system, and digestive tract. To define the molecular mechanism underlying human TMP21 gene transcription and expression, we cloned 3.3 kb of the 5' flanking region of the human TMP21 gene. We used 5'-RACE to map the major transcription start site of *TMP21 *at 43 bp upstream of the first ATG translation codon. The promoter sequence does not contain TATA or CATA boxes, typical features of most type II eukaryotic gene promoters. The promoter also does not have high GC content, such as in APP and BACE1 gene promoters [57], and does not resemble many housekeeping gene promoters [58]. Sequence analysis suggested several putative regulatory elements of the TMP21 promoter, including NFAT, HIF, CREB, YY1F, AP1 and STAT. A series of nested deletions of the 5' flanking region of the *TMP21 *promoter were subcloned into pGL3-Basic, a luciferase reporter plasmid vector, and the luciferase activity of TMP21 fragment was assayed. Our study showed that the 5' flanking region from -120 to +2 bp contains the minimal promoter region necessary for basal transcription of the human TMP21 gene. Gel shift assays confirmed that a NFAT binding site is located at -502 bp to -476 bp of the human TMP21 promoter region. Furthermore, our results showed that human TMP21 promoter activity can increase with co-expression of exogenous NFAT, and decrease after inhibition of NFAT by PDTC. Finally we showed that overexpression of NFAT could increase endogenous TMP21 mRNA and protein levels, while knockdown decreased them. These results clearly demonstrate that human TMP21 gene expression is regulated by NFAT signaling via its effect on a functional NFAT response element in the human TMP21 gene promoter region. Our study indicates that NFAT regulates TMP21 gene expression at the transcription level and the human TMP21 gene is one of the downstream target genes of NFAT signaling pathway.

Abnormal regulation of gene transcription has been implicated in the AD pathogenesis and pancreas endocrine function [16,59]. One of the pharmaceutical strategies in AD therapy is to reduce Aβ production. TMP21 protein is a new member of p24 cargo proteins and plays an important role in Aβ production in AD pathogenesis. It was reported that reduction of TMP21 can significantly increase Aβ production [38]. Our study provides new insights on the molecular mechanism underlying transcriptional regulation of the human TMP21 gene and we found that NFAT plays an important role in the regulation of TMP21 gene expression both in neuronal and non-neuronal cells. Future research will determine additional *cis*-acting elements in the TMP21 promoter responsible for its neuronal and pancreatic tissue-specific expression pattern, and how dysregulation of TMP21 expression plays a role in the pathogenesis of neuronal-endocrine disorders.

## Conclusion

In this report we have cloned a 3.3-kb fragment upstream of the human TMP21 gene. The transcription start site (TSS) of the human TMP21 gene was identified. A series of nested deletions of the 5' flanking region of the human TMP21 gene were subcloned into the pGL3-basic luciferase reporter plasmid. We identified the -120 to +2 region as containing the minimal sequence necessary for TMP21 gene promoter activity. Gel shift assays revealed that the human TMP21 gene promoter contains NFAT response elements. Expression of NFAT increased TMP21 gene expression and inhibition of NFAT by siRNA reduced TMP21 gene expression. These results demonstrated that NFAT plays a very important role in the regulation of human TMP21 gene expression.

## Methods

### Cloning of TMP21 promoter and construction of chimeric luciferase reporter plasmids

The 5'-flanking region of the human TMP21 gene was amplified by PCR from human BAC DNA NM_006827 gDNA-74712811-74722811R. The primers were designed with restriction enzymes sites compatible with the multi-cloning site of vector pGL3-basic (Promega). Various 5' flanking regions of TMP21 were cloned upstream of the luciferase reporter gene in pGL3-basic. The fragment of TMP21 was constructed from -3108 bp upstream to +225 bp downstream of transcription start site. The primers were used for deletion promoter constructs: forward, -3108: 5'-ccggtaccaaggtcaggatgttcaagaccagc, -2061: 5'-cgcgagctcctttaacagtataattatttggcctc, -1260: 5'-ccgctcgagttcaagcaattctctgcctc, -596: 5'-gctatgggacatgaaccggatgtc-3', and -120: 5'- ccgctagccctatcctttcttccc; reverse, -2061: 5'-cgcgagctcgaggccaaataattatactgttaaag, -1159 5'-gaaaccccgtctctactaaaaatac, -561: 5'- cccaagcttgttttaggggtcacctgatg, -148: 5'- gttaaagaagctttaatagaatactattgc, -57: 5'-ccctcgag tggagactggcatgtagag, -38: 5'- cccaagctttgatgccgtccgcgcc, -23: 5'- ccctcgagctcacctctgaccttc, 2: 5'-cccaagcttccggaaccggggggac, -10: 5'-ccctcgagggacccacgtgactcac, and 33: 5'- ccctcgagactcgttcaccaccg. In order to construct the longest promoter fragment of TMP21, -2061 Kpn *I *5'-cgcgagctcctttaacagtataattatttggcctc and 225: 5'- ggtcggagatctcgtacgcgccag were used to amplify -2061 to +225 bp region of TMP21 promoter gene using BAC DNA. The fragment was cloned into PGL3 basic at Kpn *I *and Bgl *II *to generate pTMP21-B, and then the fragment -3108 to -2061 was inserted in pTMP21-B at Kpn *I *to construct pTMP21-A containing the fragment of -3108 to +225 bp of TMP21 gene.

### Cell culture, transfection and luciferase assay

HEK293 cells, SH-SY5Y and N2a cells were cultured in Dulbecco's modified Eagle's medium containing 1 mM sodium pyruvate, 10% fetal bovine serum, 2 mM of L-glutamine and 50 units of penicillin and 50 ug of streptomycin (Invitrogen, Carlsbad, CA USA). All cells were cultured in an incubator at 37°C containing 5% CO_2_. Cells were seeded in 24-well plats 24 hours before transfection and grown to near 70% confluence by the day of transfection. Cells were transfected with 0.5 ug plasmid DNA per well using 1 μL Lipofectamine 2000 (Invitrogen). The Renilla (sea pansy) luciferase vector pRluc was cotransfected to normalize for the transfection efficiency of various luciferase reporter constructs. Cells were harvested at 48 hour after transfection and lysed in 50 μL 1× passive lysis buffer (Promega) per well. Firefly luciferase activities and Renilla luciferase activities were assayed using the Dual-luciferase reporter assay system (Promega). The firefly luciferase activity was normalized according to Renilla luciferase activity and expressed as relative luciferase units to reflect the promoter activity. Plasmid HA-NFAT1 expresses HA-tagged murine NFAT1 in pcDNA4 [60].

### RNA Ligase Mediated Rapid Amplification of cDNA Ends (RLM-RACE) of TMP21 gene

Total RNA was extracted from SH-SY5Y cells with TRI reagent following the manufacturer's protocol (Sigma, St Louis, MO, USA). RLM-RACE was performed according to the Instruction Manual: Poly (A) selected RNA is treated with Calf Intestine Alkaline Phosphatase (CIP) to remove free 5'-phosphates from molecules. The RNA is then treated with Tobacco Acid Pyrophosphatase (TAP) to remove the cap structure from full-length mRNA, leaving a 5'-monophosphate. A 45 base RNA Adapter oligonucleotide was ligated to the RNA samples using T4 RNA ligase. A random-primed reverse transcription reaction and nested PCR then amplifies the 5' end of a TMP21 transcript. The outer and inner primers, which were used in nested PCR, were 5'-ggtcggagatctcgtacgcgccag and 5'-cccctcgagaag gagatggcaaggaccaatc, respectively. The PCR product was inserted into pcDNA4-mycHis with BamH *I *and Xho *I*. The plasmid sequence was analyzed. The first base pair after the adapter is identified as the transcription start site of the human TMP21 gene.

### Nuclear extraction and gel shift assay

To prepare NFAT-enriched nuclear extract, HEK293 cells were transiently transfected with pHA-NFAT1 expression plasmid for 48 hours. The cells were washed with phosphate-buffered saline and harvested with five volumes of buffer A [10 mM HEPES pH7.9, 10 mM KCl, 0.1 mM EDTA, 0.1 mM EGTA, 1 mM dithiothreitol (DTT), 0.5 mM phenylmethylsulfonyl fluoride (PMSF)]. After gentle pipeting the cells were incubated on ice for 15 min. The cells were ruptured by 10 pestle strokes in a Kontes all glass Dounce tissue grinder. 10% NP40 was added to the homogenates on ice to a final NP40 concentration of 0.5% for an additional 15 min prior to 5 more strokes. Crude nuclei were collected by centrifugation at 2 000 × g for 10 min. The nuclei were washed three times with buffer A with 0.5% NP40 and resuspended in buffer C [20 mM HEPES pH7.9, 0.4 M NaCl, 1 mM EDTA, 1 mM EGTA, 1 mM dithiothreitol, 1 mM phenylmethylsulfonyl fluoride, 10% Glycerol] at 4°C for 15 min. The supernatant, which contains nuclear proteins, was collected by centrifugation at 12 000 Xg for 5 min at 4°C and stored -80°C.

Electrophoretic mobility shift assay (EMSA), also known as gel shift assay (GSA), was performed as previously described [58]. Both sense and anti-sense NFAT oligonucleotides, end-labeled with IRDye-680 (Li-COR), were annealed to generate double-stranded probes. The sequences for the sense strand probes were: consensus NFAT (Wt-NFAT): 5'-gaggaaaatttg; NFAT mutant oligonucleotides (Mt-NFAT): 5'-gaggaccctttg; TMP21-NFAT-1: 5'-tatttccttttagtaattttccatc; TMP21- NFAT-2: 5'-ccctacttttccttgtatttgga; TMP21- NFAT-3: 5'-acctcggtttcctcatctgt; TMP21- NFAT-4: 5'-gatggaaatttaaaggaaacac; TMP21- NFAT-5: 5'-catttctaggaaaataact; TMP21- NFAT-6: 5'-tctttctttccttctttcttt; TMP21- NFAT-7: 5'-gaaatctttcctcgaaattttcctct; and TMP21- NFAT-8: 5'-gtggctgtttcctattgggg. The end-labeled probes were incubated with or without nuclear extract at 22°C in binding buffer (100 mM Tris, 500 mM KCl, 10 mM DTT; pH 7.5)with 25 mM/L DTT, 2.5% Tween-20, and 1 μg poly(dI-dC)/μl in 10 mM Tris and 1 mM EDTA (pH 7.5) for 30 minutes, and the samples were analyzed on a 4% nondenaturing polyacrylamide gel. For the competition assays, the binding reaction was incubated with 10 pmol (10 times) and 100 pmol (100 times) of unlabeled competition oligonucleotides. For supershifting assay, the polyclonal TMP21 antibody raised from rabbit [38] was added to the EMSA reaction mixture.

### Chromatin Immunoprecipitation (ChIP) assay

ChIP assay was performed as described previously [61] with modification. Cross-link between protein and chromatin was achieved by addition of formaldehyde to the final concentration of 1.42% in NFAT-enriched HEK293 cells or SH-SY5Y cells for 15 min at room temperature (22°C). Cross-link was quenched with glycine at final concentration of 125 mM for 5 min at room temperature. Cells were then harvested in cold PBS and lysed with IP buffer containing 150 mM NaCl, 50 mM Tris-HCl (pH 7.5), 5 mM EDTA, NP-40 (0.5% vol/vol), Triton X-100 (1.0% vol/vol), and 0.5 mM phenylmethylsulfonyl fluoride (PMSF). The nuclear pellets were isolated by centrifugation at 12,000*g *for 1 min, and resuspended with the IP buffer. The chromatin was then sheared by sonication on ice. For isolation of NFAT binding complex, the chromatin solution was incubated with NFAT antibody (Santa Cruz) or equal volume of PBS overnight at 4°C. After immunoprecipitation, cross-link was reversed by adding Chelex100 and boiling beads for 10 min. The supernatant containing DNA fragments was isolated by centrifugation at 12,000*g *for 1 min, which was then used as template for PCR analysis of the chromatin fragment. "Primers" were used to amplify NFAT putative consensus binding site (need to confirm with Fang) located on the TMP21 promoter region. Actin was used as internal control.

### Quantitative RT-PCR

Total RNA was isolated from cells using TRI reagent (Sigma). ThermoscripTM RT-PCR system (Invitrogen) was used to synthesize the first strand cDNA using 5 μg of total RNA as template following the manufacurer's instructions. The newly synthesized cDNA templates were further amplified by Platinum Taq DNA polymerase (Invitrogen) in a 50 μl reaction. The human TMP21 gene specific primers 5'-cgggatccgccaccatgtctggtttgtctggcccac forward, and 5'-ggaattcctcaatcaatttcttggccttg reverse, were used to amplify a 660-bp fragment of the TMP21 coding region. β-actin was used as an internal control. β-actin gene-specific primers were: forword: 5'- cgaggatccggacttcgagcaagagatgg; reverse: 5'- cagtctagagaagcatttgcggtggacg. All PCR products were analyzed on 1.5% agarose gels.

### Immunoblotting

Cell lysates were resolved by 16% Tris-glycine sodium dodecyl sulfate-polyacryamide gel electrophoresis and immunblotting analysis was performed as described previously [38]. A rabbit anti-TMP21 polyclonal antibody T21 raised against the TMP21 protein was used to detect TMP21 expression [38]. Internal control β-actin expression was analyzed with monoclonal anti-β-actin antibody AC-15 (Sigma).

## Competing interests

The authors declare that they have no competing interests.

## Authors' contributions

SL and WS conceived the study and designed the experiments, SL, SZ, KB, FC, WZ and JM performed the experiments and evaluated the data, and KX provided reagents. SL, SZ, KB and WS wrote the paper. All authors have read and approved the final manuscript.
